# Procyanidin-B1-Enriched *Cyperus esculentus* Extract Regulates Anti-Inflammatory Pathways in Chicken Macrophages Cells Through Transcription Factor STAT2 and HIF1A

**DOI:** 10.3390/ani15233469

**Published:** 2025-12-02

**Authors:** Siqi Niu, Fanghong Zhang, Juan Li, Jianwu Wang, Tinghua Huang, Min Yao

**Affiliations:** 1 College of Animal Science and Technology, Yangtze University, Jingzhou 434025, Chinathua45@yangtzeu.edu.cn (T.H.); 2 College Chemistry, Xiangtan University, Xiangtan 411105, China; 3 College of Agriculture, Yangtze University, Jingzhou 434025, China

**Keywords:** *Cyperus esculentus* extract, anti-inflammatory, transcription factors, macrophage polarization, HIF1A

## Abstract

Procyanidin B1 (PB1), a polyphenol abundant in *Cyperus esculentus* stems and leaves extract (CELE), exhibits antioxidant and anti-inflammatory activities, though its mechanisms are not fully understood. This study investigated CELE’s effects in chickens and LPS-stimulated HD11 macrophages. Chickens fed CELE showed increased blood levels of SOD, GSH-Px, TNF-α, IL-1β, IL-6, and IL-10, while MDA decreased. RNA-seq of LPS + PB1 vs. LPS-treated cells identified 696 differentially expressed genes enriched in inflammation and antioxidant pathways. Analysis indicated 120 transcription factors (TFs) may regulate these changes, with FOSL1, HIF1A, and STAT2 significantly downregulated. In HD11 cells, PB1 reduced expression of HIF1A/STAT2-target genes (e.g., HMGA2, EPSTI1), lowered IL-1β, IL-6, and ROS, and shifted macrophage polarization from M1 to M2. PB1’s effects were enhanced by an HIF1A inhibitor but reversed by a STAT2 activator. These findings support PB1 and CELE as potential feed additives for livestock.

## Simple Summary

Procyanidin B1 (PB1) is a natural compound found in the stems and leaves of the plant *Cyperus esculentus*, known for its health benefits. This study explored how an extract from this plant (CELE) and PB1 can improve health in chickens and immune cells. We found that feeding chickens a diet with CELE boosted their antioxidant levels and modulated their immune responses. In lab studies using immune cells challenged with a toxin (LPS), PB1 showed strong anti-inflammatory effects. It worked by targeting key proteins (specifically HIF1A and STAT2), which in turn reduced inflammation and harmful molecules. Furthermore, PB1 helped shift the immune cells from a proinflammatory state to a healing state. Our findings suggest that both PB1 and the *Cyperus esculentus* extract are promising natural options for use as feed additives to enhance animal health.

## 1. Introduction

Procyanidins are a group of flavonoid polymers that are widely found in plants, formed by the polymerization of monomers such as catechin and epicatechin (flavan-3-ols). The benzodihydropyran (flavan-3-ol) ring, multiple free phenolic hydroxyl groups, and chiral carbon atoms (C-2 and C-3) in their molecular structure endow these compounds with strong electron-donating capacity and free radical scavenging ability. These properties serve as the structural basis for their antioxidant bioactivity [1,2]. Grape seed procyanidin extract has been shown to enhance the total antioxidant capacity in cells of obese rats by suppressing the activity of antioxidant enzymes, including glutathione peroxidase, and alleviating obesity-induced oxidative stress [3]. The administration of grape seed procyanidins prevents oxidative stress and alleviates apoptosis and autophagy instigated by H_2_O_2_, aflatoxin B_1_, and an array of chemical agents. This regulatory effect is achieved through the activation of SIRT1 and FOXO1 transcription, as well as the NF-κB and Nrf2 signaling pathways [4,5,6].

A substantial body of research has demonstrated that procyanidins have significant anti-inflammatory potential. The administration of procyanidins derived from grape seeds has been demonstrated to alleviate pain associated with gout by suppressing the NLRP3 inflammasome [7]. In addition, these procyanidins have been shown to ameliorate inflammation in insulin-resistant mice via the NF-κB and NLRP3 inflammasome pathways [8]. Type A procyanidin has been shown to target the NF-κB, MAPK, and Nrf2/HO-1 pathways, leading to the downregulation of proinflammatory factor release, ROS, and NO, and the protection against lipid peroxidation in Raw464.7 cells [9,10]. Type B Procyanidin has been demonstrated to alleviate oxidative stress and inflammatory damage induced by cadmium exposure in uterine sepsis [11]. In addition to these properties, procyanidins have been shown to possess the capacity to protect intestinal barriers and exhibit anticancer properties. For instance, procyanidin has been shown to regulate NETosis and to impede the growth and proliferation of liver cancer cells [12]. Studies demonstrated that dietary supplementation with grape seed procyanidins improved the integrity of the intestinal barrier, increased the relative abundance of beneficial bacteria, decreased the level of potentially pathogenic bacteria, and modulated lipid metabolism [13,14]. Plant-derived procyanidins have been proposed as a feed additive to improve growth performance and enhance disease resistance in livestock and poultry [15,16].

Procyanidin B1 (PB1), a representative flavan-3-ol dimer, is widely distributed in plants such as grapes, cocoa, apple peel, cinnamon, and berries [17]. Recent studies have revealed that *Cyperus esculentus* leaves contain a relatively high content of procyanidin B1 with few isomers [18]. Procyanidin B1, like other procyanidin compounds, has been shown to possess antioxidant, anti-inflammatory, and potentially antineoplastic properties [19]. However, the number of studies that have focused on the transcription factors that it can target is very limited. In this study, procyanidin B1 in *Cyperus esculentus* stem and leaf extract (CELE) was quantified using a Procyanidin B1 standard. The extract was then incorporated into chicken diets to investigate its effects on growth performance, blood antioxidants, and anti-inflammatory factors. Additionally, the transcriptome changes of procyanidin B1 standard in the chicken macrophage-like HD11 cells inflammation model were determined. The identification of the key transcription factors involved in the regulation of transcriptome changes was achieved through the utilization of a software package called FLAVER [20,21], which employed a weighted Kendall’s ranking correlation algorithm to test the significance of the potential of transcription factor binding and extend the differential expression of the target genes. Subsequent experiments corroborated the finding that procyanidin B1 exerts its effects on anti-inflammatory transcription factors, specifically STAT2 and HIF1α. The findings of this study offer significant insights into the anti-inflammatory mechanisms of procyanidin B1 and furnish a data set for further investigation of the potential of *Cyperus esculentus* stems and leaves in the improvement of chicken health.

## 2. Materials and Methods

### 2.1. Chemicals and Reagents

RPMI 1640 medium (11875085), fetal bovine serum (FBS, A3161001C), and penicillin–streptomycin (15140148) were obtained from Gibco (Gaithersburg, MA, USA). The following reagents were procured from Beyotime Biotechnology (Shanghai, China): Lipopolysaccharide (LPS) derived from *Escherichia coli* O111:B4 (S1735), Reactive Oxygen Species (ROS) detection kit (S0033S), Superoxide Dismutase (SOD) assay kit (S0101S), Lipid Peroxidation MDA Assay Kit (S0131S), and Total Glutathione (GSH-Px) Assay Kit (S0052). Enzyme-linked Immunosorbent Assay (ELISA) kits for interleukin-1β (IL-1β, CSB-E11230Ch), interleukin-6 (IL-6, CSB-E08549Ch), tumor necrosis factor-α (TNFα, CSB-E11231Ch), and interleukin-10 (IL-10, CSB-E12835C) were obtained from Huamei Biological Engineering Research Institute (Wuhan, China). The MiniBEST Universal RNA Extraction Kit (9767), the PrimeScript™ RT reagent Kit (RR037A), and the TB Green^®^ Premix Ex Taq™ II FAST qPCR kit (CN830A) were obtained from Takara (Beijing, China). Procyanidin B1 standard (B21616, CAS No. 20315-25-7, ≥95%) was obtained from Shanghai Yuanye Bio-Technology Co., Ltd. (Shanghai, China). Retinoic acid (RA), an activator of STAT2 (sc-200898), and a hypoxia-inducible factor-1α (HIF-1α) inhibitor (HIF1Ai, sc-205346) were obtained from Santa Cruz Biotechnology, Inc. (Dallas, TX, USA). The FITC-conjugated anti-chicken MHC-II antibody (ab24882) was obtained from Abcam, while the PE-conjugated rabbit anti-human CD163 antibody (A28196) was obtained from ABclonal Co., Ltd. (Wuhan, China). The chicken macrophage-like HD11 cell line (HD11) was kindly provided by Dr. Jiao Song from the College of Life Sciences at Yangtze University. The Primer sequences for Real-time PCR were synthesized by Sangon Biotech (Shanghai, China).

### 2.2. Extraction and Quantification of PB1 in Cyperus esculentus Stems and Leaves

The stems and leaves of *Cyperus esculentus*, collected in August, were used for procyanidin extraction. After extraction, PB1 was quantified using an HPLC-UV system with a PB1 standard for calibration. The extraction and quantification methods were adapted from a previously Ma and Yonekura’s procedure [22,23]. The final extracts were subjected to vacuum evaporation before they were added to the feed, finally obtained solid powder enriched by anthocyanin B1. In this study, 100 mg/kg solid powder was mixed into the feed diet based on the quantitative results and the concentration adapted from the previous feeding trial [13].

### 2.3. Chicken Feeding Test for CELE

A total of sixty healthy 1-day-old white Leghorn chickens (30 males and 30 females) of uniform body weight were used in this study. The experiment period lasted 42 days, and the chickens were fed a standard chicken diet from day 1 to day 7. On day 7, the chickens were divided into three dietary treatment groups in a completely randomized design, with five replicates (pens) per group. Each replicate (pen) contained four birds (two males and two females), resulting in a total of 20 chickens per treatment group. At the time of grouping, it was ensured that the average initial body weight showed no statistically significant differences across the treatment groups.

The management method for chickens in this study is the same as the method described in our previous article on the chicken feeding experiment of SeMC [24]. It was summarized as follows: The relative humidity was consistently maintained at 40 to 60%. The room temperature within the feeding room was maintained at 32 to 34 °C, with weekly reductions of 2 °C until it reached the final range of 22 to 24 °C. Waste was cleaned out every day, and the air in the house was kept fresh.

The control and LPS groups were fed a standard diet until the end of the experiment. The CELE + LPS group was fed a standard diet containing 100 mg/kg PB1 until the end of the experiment [13]. The nutritional composition of the experimental diets matched that of a standard chicken diet published previously [24]. On day 42, the LPS and PB1 + LPS groups received a single intraperitoneal injection of LPS (1 mg/kg), while the control group received an equivalent volume of PBS. Body weight was measured on days 7 and 42 to calculate the average daily gain (ADG). Total feed intake was recorded, and the average daily feed intake (ADFI) was subsequently computed. The feed conversion ratio (FCR) was calculated as feed intake divided by body weight gain. Following a six h LPS challenge, 4 mL of blood was collected from the wing vein of each bird. Two milliliters of the blood sample were transferred into an EDTA-coated anticoagulant tube for subsequent real-time PCR and ELISA analysis of inflammatory cytokines. The remaining 2 mL was allowed to clot, and serum was isolated by centrifugation at 2000× *g* for 10 min at 4 °C. The serum was then stored at −80 °C for subsequent antioxidant capacity analysis. All animal experiments in this study were conducted in compliance with the Regulations for the Administration of Experimental Animals issued by the China Science and Technology Commission (No. 2006-398) and were reviewed and approved by the Animal Ethics Committee of Yangtze University (No. 2024-041, 03/01/2024, Jingzhou, China).

### 2.4. Cell Treatment and LPS Stimulation

HD11 cells were cultured as described previously [24]. The HD11 cells were then seeded into 6-well plates at a density of 3 × 10^6^ cells per well and allowed to adhere for 12 h. Samples (*n* = 3) for RNA-seq were collected from four treatment groups with three replicates each. The treatment groups included PB1, PB1 + LPS, LPS, and Control (PBS, CTR). Samples for subsequent assays, including ROS detection, inflammatory cytokine ELISA, macrophage polarization, and analysis of transcription factors and their target gene expression, were collected from two independent experiment groups with each have five treatment: (i) Control, lipopolysaccharide (LPS), PB1 + LPS, LPS + retinoic acid (RA), and PB1 + LPS + RA; (ii) Control, lipopolysaccharide (LPS), PB1 + LPS, LPS + HIF1Ai, and PB1 + LPS + HIF1Ai. Cells were incubated with PB1 (100 μg/mL) and either RA or HIF1A (5 μM) for 12 h, then exposed to LPS (100 ng/mL) for another 12 h. The concentration of procyanidin B1 was confirmed to be suitable using an MTT cell viability assay. In brief, HD11 Cells were seeded in each well of 96-well plates at a density of 10,000/well in 200 μL complete RPMI-1640 medium and cultured at 37 °C. After 12 h, The cells were exposed to a series of PB1 concentrations (0, 25, 50, 100, and 200 μg/mL) in combination with 100 ng/mL of LPS. After 24 h, 10 μL of MTT solution (5 mg/mL in PBS) were added and incubated at 37 °C for four h. Then, the medium was removed, and 150 μL of DMSO was added to each well. Following vibrating on a shaker for 10 min, the plates were measured for absorbance at 570 nm wavelength. The relative viability of HD11 cells was calculated using the following formula: Cell relative viability (%) = (A_treatment_/A_control_) × 100. The highest concentration of PB1 with cell viability greater than 95% was used for subsequent cell experiments.

### 2.5. High-Throughput Sequencing

Cells were lysed with 1 mL of Trizol reagent and immediately snap-frozen in liquid nitrogen. The frozen samples were subsequently transported on dry ice to the DNA sequencing facility for RNA-seq analysis. Sequencing libraries were prepared using the Illumina TruSeq RNA Sample Preparation Kit (Illumina Inc., San Diego, CA, USA) following the manufacturer’s instructions. The Sequencing was performed on an Illumina HiSeq 2500 instrument (Illumina Inc., USA) using a single-read sequencing method. The raw data were then filtered according to the manufacturer’s recommendations to remove low-quality reads and obtain high-quality clean data. The Hisat2 v2.2 software [25] was employed to map clean reads to the reference genome (GRCg7b), which was extracted from the NCBI genome database [26]. The calculated read count per gene was estimated by Htseq-count [27] and used for comparing the difference in gene expression among samples. The DESeq2 R package was used for the calculation, and the criteria for differentially expressed genes were defined as FDR (false discovery rate) ≤ 0.05 and FC (fold change) ≥ 1.5 or ≤0.67. The data generated in this study are available in the NCBI GEO database under accession number GSE309607.

### 2.6. Identification of Key Transcription Factors

The identification of key transcription factors (TFs) regulating the differentially expressed genes was conducted using a multi-step analytical strategy. First, a gene set containing putative targets of each TF and a separate list of differentially expressed genes were compiled. Correlation analysis was then performed between these sets to infer TF–gene regulatory relationships. Transcription factor binding site (TFBS) data were predicted using GRIT-2.0, which integrates both binding affinity (Jindex) and cross-species conservation information within a mixed Student’s *t*-test framework [28]. As established by Huang et al. [20], the Jindex quantifies the maximum of repeated averaging of log likelihood ratios (LLRs), which are indicative of the potential presence of a motif at a specific location in a sequence. The correlation analysis was performed using the FLAVER v2.0 software package. Based on the approach introduced by Yao et al. [20,21], this study identified key transcription factors from transcriptome data by assessing the statistical significance of the correlation between the ranked gene set and the ordered gene list.

### 2.7. Real-Time Polymerase Chain Reaction (Real-Time PCR) Analysis

Total RNA was extracted from HD11 cell samples using the MiniBEST Universal RNA Extraction Kit. The isolated RNA was reverse-transcribed into cDNA using a PrimeScript™ RT reagent Kit. The primer sequences for the relevant transcription factors and their target genes are listed in Supplemental Table S1. Real-time PCR was performed using a TB Green^®^ Premix Ex Taq™ II FAST qPCR on a BIORAD Cycler. Gene expression was quantified using the 2^−ΔΔCT^ method and normalized to the expression of GAPDH.

### 2.8. Antioxidant Enzyme Assay

The activities of antioxidant enzymes (SOD and GSH-Px) and the concentration of MDA in chicken serum samples were measured using commercially available kits according to the manufacturer’s instructions, with appropriate dilution applied as required.

### 2.9. Enzyme-Linked Immunosorbent Assay

The concentrations of proinflammatory (TNF-α, IL-1β, IL-6) and anti-inflammatory (IL-10) cytokines in chicken plasma and HD11 cell culture supernatants were measured using ELISA kits that were specific to chickens. Samples were diluted and processed according to the manufacturer’s protocol.

### 2.10. ROS Detection

HD11 cells were gently scraped from 6-well plates and centrifuged to remove the medium. The cell pellets were then incubated with 1 mL of 10 μM DCFH-DA at 37 °C for 30 min. Following three washes with PBS, intracellular ROS levels were measured using a BD FACS Melody flow cytometer. ROS production was quantified as the percentage of DCF-positive cells within the gated population.

### 2.11. Polarization Analysis of HD11 Cells

Flow cytometry was performed to analyze the expression of M1 and M2 macrophage markers. HD11 cells were harvested by scraping, washed three times with PBS, and then blocked with 2% chicken IgY on ice for 20 min. After washing, the cells were stained with FITC-conjugated anti-chicken MHC-II (1:200) and PE-conjugated anti-human CD163 (1:50) at 4 °C for 30 min in the dark. The cells were then fixed in 250 μL of fixation buffer at room temperature for 30 min in the dark, washed, and finally resuspended in 400 μL of PBS for flow cytometric analysis. Results were quantified as the percentage of FITC-positive or PE-positive cells within the gated population.

### 2.12. Statistical Analysis

Data from mRNA quantification, oxidase activity, cytokine, ROS, and macrophage polarization assays were analyzed by a one-way ANOVA, followed by Duncan’s post hoc test for multiple comparisons, using R software (version 4.2).

## 3. Results

### 3.1. Effects of ECLE on Growth Performance, Blood Antioxidant Capacity, and Inflammatory Cytokines in Broiler Chickens

**Figure 1:**
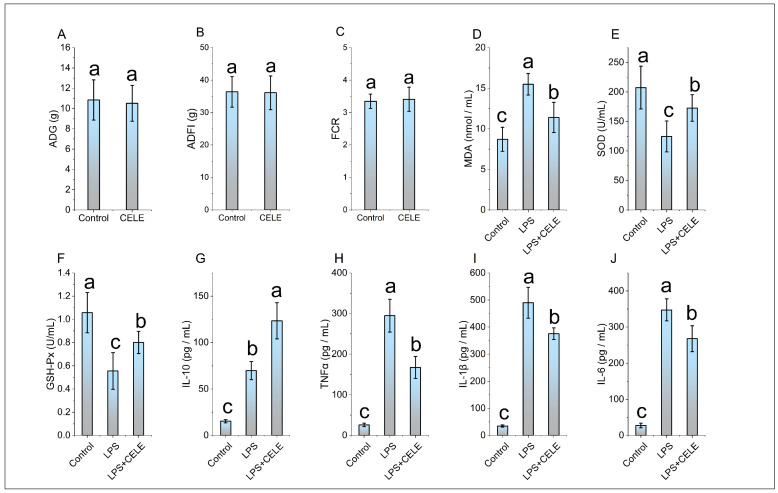
The effects of CELE on weight gain, blood antioxidant parameters, and blood inflammatory cytokines in broiler chickens. “Weight Gain” is defined as the average value of body weight at 42 days (g) minus body weight at 7 days (g) for the broilers in each treatment group. (**A**) Average daily gain (ADG), (**B**) Average daily feed intake (ADFI), (**C**) Average feed conversion ratio (FCR), (**D**) Serum malondialdehyde (MDA) content, (**E**) Serum superoxide dismutase (SOD) activity, (**F**) Serum glutathione peroxidase (GSH-Px) activity, (**G**) Plasma interleukin-10 (IL-10) level, (**H**) Plasma tumor necrosis factor-alpha (TNFα) level, (**I**) Plasma interleukin-1 beta (IL-1β) level, (**J**) Plasma interleukin-6 (IL-6) level. The same shoulder label letter indicates no significant difference (*p* > 0.05), different lowercase letters indicate significant difference (*p* < 0.05).

In this study, the content of PB1 in CELE was found to be 89.5%. The HPLC chromatograms of PB1 from the extract and the PB1 standard are shown in Figure S1 (Supplementary Material). The feeding test results demonstrated that there was no significant difference in ADG, ADFI, and FCR between the chickens supplemented with CELE and the control group (see Figure 1A–C). These results suggest that CELE does not impact the normal growth of chickens. The antioxidant capacity and cytokine levels were measured in plasma samples, with results shown in Figure 1D–F. Consistent with previous findings, serum MDA levels were significantly elevated in LPS-challenged chicks compared to controls, while activities of the antioxidant enzymes SOD and GSH-Px were markedly reduced (*p* < 0.05), reflects a typical process of antioxidant protection stress. Serum levels of SOD and GSH were significantly higher in the CELE + LPS group compared to the LPS-only group (*p* < 0.05), indicating that CELE enhanced the antioxidant defense against LPS-induced oxidative stress. Serum levels of IL-1β, IL-6, TNFα, and IL-10 were markedly elevated in LPS-treated chickens, reaching approximately 14-, 12-, 11-, and 4-fold those of control chickens, respectively. These results collectively demonstrate that LPS successfully established a model of inflammatory syndrome in chickens. Serum levels of IL-1β, IL-6, and TNFα were elevated in chickens pretreated with CELE compared to controls, yet remained significantly lower (*p* < 0.05) than those in the LPS-only group—specifically, decreasing to 76%, 77%, and 57% of LPS-induced levels, respectively (Figure 1H–J). In contrast, IL-10 levels were significantly higher in CELE-pretreated chickens than in those receiving LPS alone (Figure 1G).

### 3.2. PB1-Mediated Transcriptome Reprogramming of HD11 Cells

**Figure 2:**
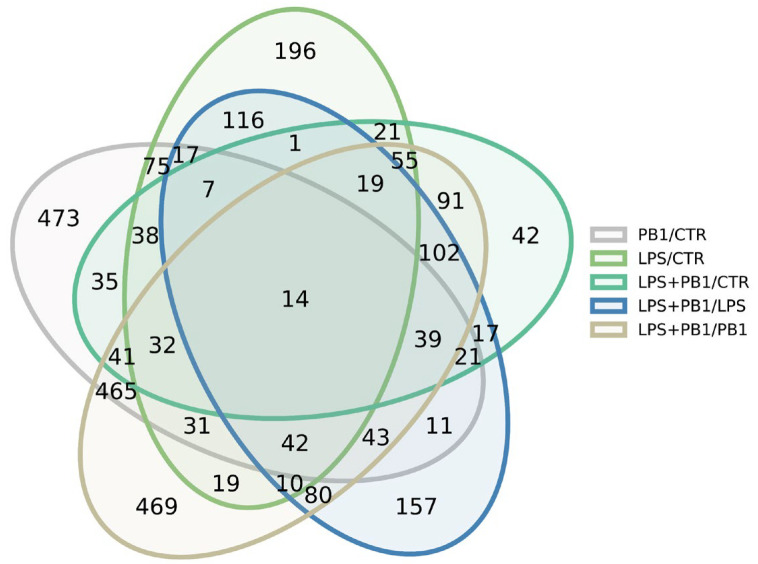
Venn diagram showing the number of differentially expressed genes in PB1-treated HD11 cells, as identified by RNA-seq.

RNA sequencing of PB1- and LPS-treated HD11 cells yielded an average of 3.2 million reads per sample, which were mapped to 12,351 annotated chicken transcripts. Analysis revealed 1068 significantly differentially expressed transcripts (FDR < 0.05) between LPS-treated and control groups. These were predominantly enriched in the Toll-like receptor signaling pathway and bacterial infection-related genes, such as TLR4, JUN, and TNFSF10. A total of 1398 transcripts were significantly differentially expressed (FDR < 0.05) between the PB1-treated and control groups. Among these, inflammation-related genes—such as TSPAN18, BST1, HIF1A, FOXF1, RBPJ, CAMK2N1, and CMKLR1—and antioxidant-related genes—including ABCC9, LOXL3, and SQOR—were notably represented. A total of 1060 transcripts were significantly differentially expressed (FDR < 0.05) between the LPS + PB1 and control groups. These included cytokine-related genes such as CRLF1, TIMP3, ADRA2A, BMP4, KIT, and TNFRSF19. A total of 1110 transcripts were significantly differentially expressed (FDR < 0.05) between the LPS + PB1 and LPS-only groups. These included inflammation-related genes (e.g., KCNJ8, CCN3, AGTR1, F2R, APOD) and antioxidant-related genes (e.g., ABCC9, APOD, SOX9). Furthermore, 1999 transcripts were differentially expressed between the LPS + PB1 and PB1-only groups. Among these, numerous genes involved in T cell activation were identified, including CD74, CBLB, NFKBIZ, CD47, and GNAI1. The Venn diagram in Figure 2 shows the number of differentially expressed genes (DEGs) for each comparison, and the complete gene lists are provided in Supplemental Table S2.

A substantial body of research has demonstrated the robust antioxidant properties of procyanidins. In this study, although no significant difference was observed in the mRNA expression level of the antioxidant transcription factor NFE2L2 (also known as NRF2) between the LPS + PB1 and LPS groups, representative target genes regulated by NFE2L2, including GCLM (FDR < 1 × 10^−5^) and SOD2 (FDR < 1 × 10^−10^) were significantly up-regulated in the LPS + PB1 group compared to the LPS group. These results suggest that NFE2L2 plays an important role in PB1-mediated antioxidant in HD11 cells, and the regulation of NFE2L2 by PB1 may occur at the post-transcriptional or protein level. NFE2L2 (NRF2) is a key transcription factor central to cellular defense against oxidative stress and inflammation. It regulates the expression of antioxidant and phase II detoxification enzymes—such as glutathione S-transferase (GST), NAD(P)H quinone oxidoreductase 1 (NQO1), and heme oxygenase-1 (HO-1)—by binding to the antioxidant response element (ARE). NRF2 also helps maintain redox homeostasis by upregulating enzymes involved in glutathione (GSH) synthesis and modulating the activity of antioxidant enzymes, including superoxide dismutase (SOD) and catalase (CAT). In this study, multiple NRF2 target genes were up-regulated in the PB1 + LPS group compared to the LPS-only group, indicating a potential role in PB1-mediated antioxidant responses. These findings are consistent with previous reports by Wei Gao et al. [29].

### 3.3. Key Transcription Factors Regulated by PB1 in LPS-Stimulated HD11 Cells

**Figure 3:**
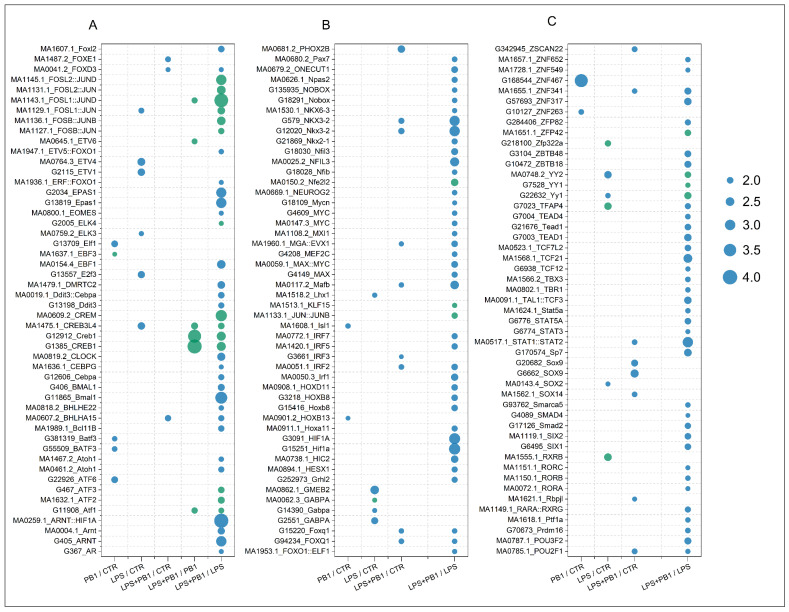
FLAVER analysis results of the transcriptome data from chicken layers treated with procyanidin B1. The dots represent the Log10(FDR) values from the FLAVER analysis results, with green indicating a positive correlation between transcription factor binding sites and target gene expression, and blue indicating a negative correlation. Results of the significant transcription factors are shown in plots (**A**–**C**) arranged in alphabetical order.

A total of 9, 17, 20, 6, and 119 transcription factors were identified in the PB1/CTR, LPS/CTR, LPS + PB1/CTR, LPS + PB1/PB1, and LPS + PB1/LPS comparison groups, respectively (FDR < 0.05, Figure 3). Specifically, 62 transcription factors showed significant correlations (FDR < 0.05) in the LPS + PB1/LPS treatment group. The most significant transcription factors across the groups included FOSL1: JUND, CREB1, ZNF467, STAT2, and HIF1A. Furthermore, the variations in target gene expression were positively correlated with the binding site penalty scores for FOSL1: JUND and CREB1 in both the LPS + PB1/LPS and LPS + PB1/PB1 groups. In contrast, a negative correlation was observed between the variations in target gene expression and the penalty scores of STAT2 binding sites in the LPS + PB1/LPS and LPS + PB1/CTR groups. Similarly, the variations in target gene expression were negatively correlated with the penalty scores of HIF1A binding sites in the LPS + PB1/LPS group. Notably, in the LPS + PB1/LPS group, HIF1A expression was downregulated by 1.98-fold (FDR < 5 × 10^−3^), STAT2 was downregulated by 1.37-fold (FDR < 7 × 10^−6^), while FOSL1 expression was upregulated by 2.12-fold (FDR < 1 × 10^−32^). These results suggest that these transcription factors may represent key targets of PB1.

### 3.4. The Expression Levels of Transcription Factors and Their Target Genes

**Figure 4:**
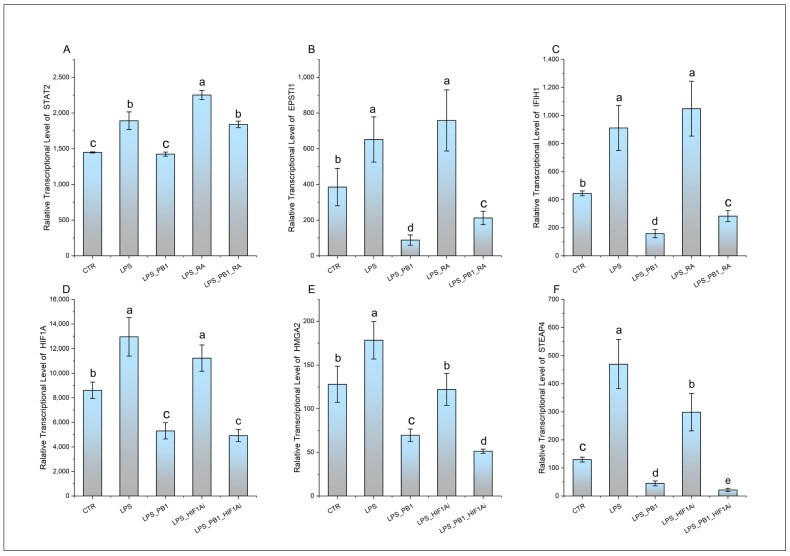
The transcription levels of STAT2, HIF1A, and their target genes in HD11 cells treated with PB1 and LPS. The height of bars indicates the relative transcriptional levels of genes. Bars bearing different letters indicate statistically significant differences (*p* < 0.05). (**A**–**F**): STAT2, EPSTI1, IFIH1, HIF1A, HMGA2, and STEAP4.

The expression of transcription factors STAT2 and HIF-1α, along with their respective target genes EPSTI1, IFIH1, HMGA2, and STEAP4, is presented in Figure 4. While LPS treatment significantly enhanced the transcription of STAT2 and its target genes (EPSTI1 and IFIH1), this effect was suppressed by PB1 co-treatment, whereas RA treatment markedly increased their expression. Similarly, LPS stimulation notably increased the transcriptional levels of HIF-1α and its target genes HMGA2 and STEAP4. This enhanced expression was significantly suppressed by co-treatment with either a HIF-1α inhibitor (HIF1Ai) or PB1. Moreover, the combination of PB1 and HIF1Ai resulted in a more profound suppression of HMGA2 and STEAP4 upregulation.

### 3.5. Effect of PB1 on Cytokine Production in HD11 Cells

**Figure 5:**
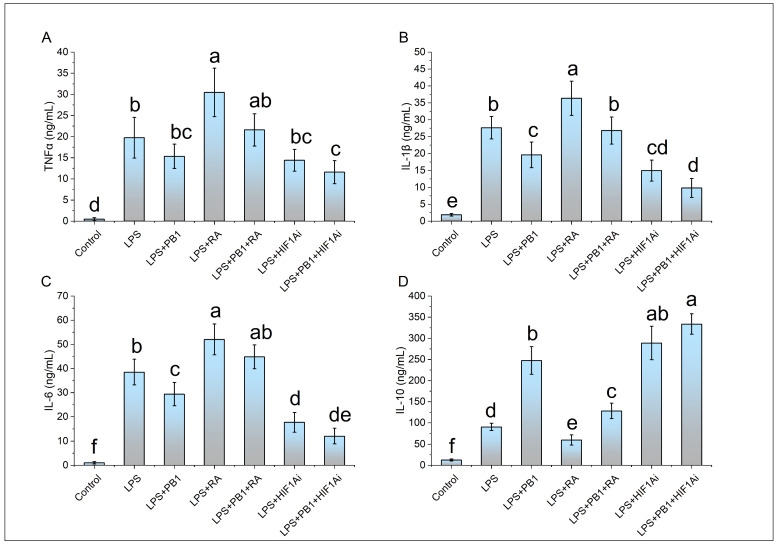
The effects of PB1, RA, and HIF1Ai on inflammatory cytokines in LPS-stimulated HD11 cells. (**A**) Tumor necrosis factor-alpha (TNFα) level, (**B**) Interleukin-1 beta (IL-1β) level, (**C**) Interleukin-6 (IL-6) level, (**D**) Interleukin-10 (IL-10) level. The same shoulder label letter indicates no significant difference (*p* > 0.05), different lowercase letters indicate significant difference (*p* < 0.05).

ELISA results demonstrated that the levels of proinflammatory cytokines (IL-1β, IL-6, and TNFα) as well as the anti-inflammatory cytokine IL-10 were significantly elevated in LPS-stimulated HD11 cells compared to controls. In the PB1 + LPS treatment group, the levels of IL-1β and IL-6 were significantly downregulated, reaching only 71% and 76% of those in the LPS-only group, respectively. Although TNFα levels also decreased in PB1 + LPS-treated HD11 macrophage-like cells, the reduction was not statistically significant. These in vitro findings are largely consistent with the results from animal serum tests. Furthermore, RA treatment notably enhanced the production of IL-1β, IL-6, and TNFα in HD11 cells challenged with either LPS alone or the combination of PB1 and LPS, while significantly suppressing IL-10 levels. In contrast, HIF1Ai treatment led to a substantial decrease in the expression of IL-1β, IL-6, and TNFα, and an increase in the expression of IL-10, under identical conditions (Figure 5).

### 3.6. Effects of PB1 on ROS Production in HD11 Cells

**Figure 6:**
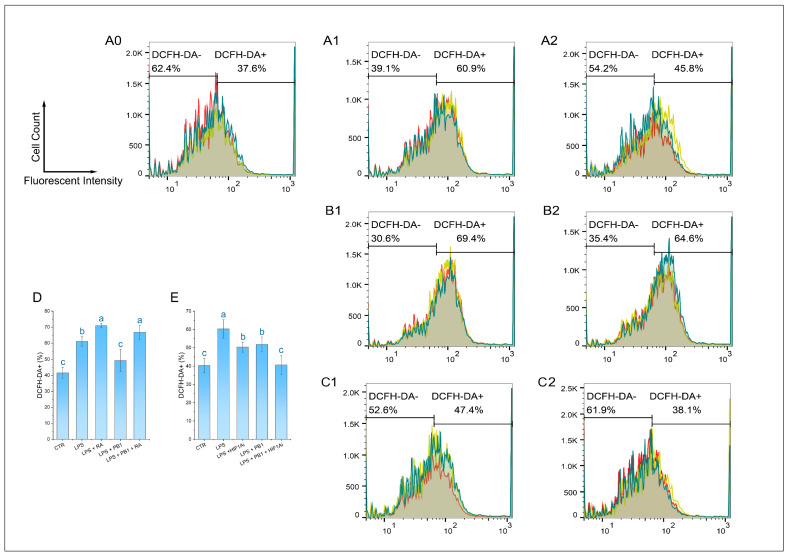
Effect of PB1 on ROS production in LPS-challenged HD11 Cells. (**A0**–**A2**,**B1**,**B2**,**C1**,**C2**) are the representative single-channel flow cytometry histograms. The results are presented as histograms of the relationship between fluorescence intensity (X-axis, DCF) and cell count (Y-axis). The results from the Control group, the LPS-treated Control group, and the LPS + PB1-treated group are displayed in Column (**A0**–**A2**). (**B**,**C**) present the results of treatment in the presence of RA and in the presence of HIF1Ai, respectively. A vertical gate separates the two populations, with percentages labeled for DCF-DA^-^ and DCF-DA^+^ subsets. Histograms presented the fluorescence distribution across three replicates (different colors), with shaded areas denoting overlapping populations. (**D**,**E**) are bar graphs of ROS levels in HD11 cells from different treatment groups. Bars bearing different letters indicate statistically significant differences (*p* < 0.05).

The ROS level of HD11 cells was detected by a DCFH-DA reagent. The results demonstrated that the proportion of DCF-DA+ cells in the LPS treatment group was 61.13 ± 2.96%, which was significantly higher than that in the control group. In contrast, the proportion of DCF-DA+ cells in the LPS + PB1 treatment group was 49.23 ± 6.92%, which was significantly lower than that in the LPS treatment group (*p* < 0.01). The proportions of DCF-DA+ cells in the LPS + RA group and the LPS + PB1 + RA group were found to be 70.93 ± 1.34% and 66.8 ± 4.43%, respectively. In comparison with the control group that did not receive RA treatment, RA treatment led to a significant increase in the proportion of DCF-DA+ cells in both the LPS group and the PB1 + LPS group (*p* < 0.05) (Figure 6D). The analysis of flow histograms revealed a statistically significant shift in the cell population towards the left (decreased fluorescence) in the PB1-treated group. In contrast, the distribution exhibited a shift towards the right in the LPS- and RA-treated groups, consistent with the quantitative results. This finding suggests that PB1 may attenuate ROS accumulation in LPS challenged HD11 cells, potentially by acting on the STAT2 pathway. Similarly, HIF1Ai treatment led to a substantial decrease in the proportion of DCF-DA+ cells in both the LPS- and PB1 + LPS-treated groups (*p* < 0.05) (Figure 6E). The more pronounced reduction observed in the PB1 co-treated group suggests that PB1 may further attenuate ROS accumulation in HD11 macrophage-like cells, potentially through the HIF-1α pathway.

### 3.7. Levels of M1/M2 Polarization Markers in HD11 Cells

**Figure 7:**
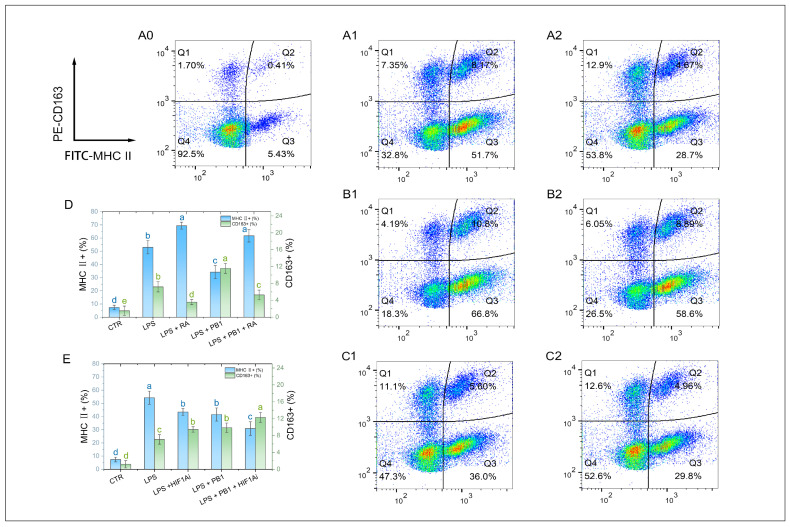
The effects of PB1 on M1/M2 polarization of HD11 cells. (**A0**–**A2**,**B1**,**B2**,**C1**,**C2**) are the representative flow cytometry analyses of the percentage of MHC II^+^ macrophages and CD163^+^ macrophages in HD11 cells. The X-axis indicates the fluorescence intensity of MHC II^+^ cells, while the Y-axis denotes the fluorescence intensity of CD163^+^ cells. A quadrant gate is employed to subdivide the cell populations into four distinct sections, with Q2 (**top right**) denoting double-positive cells, Q3 (**bottom right**) representing M1 cells, Q1 (**top left**) representing M2 cells, and Q4 (**bottom left**) classifying unpolarized cells. Columns (**A0**–**A2**) present samples from the untreated control group, the LPS-treated group, and the LPS + PB1-treated group, respectively. The rows labeled (**B**,**C**) correspond to the presence of RA or HIF1Ai co-treatment, respectively. (**D**,**E**) are bar graphs for the percentages of MHC II^+^ macrophages (blue) and CD163^+^ macrophages (green) in HD11 cells from different treatment groups. Bars bearing different letters indicate statistically significant differences (*p* < 0.05).

M1-type macrophages were labeled with MHC II, and M2-type macrophages were labeled with CD163. Flow cytometry analysis showed that 12 h LPS stimulation significantly altered the polarization state of HD11 cells. Compared to the control group, the proportions of MHC II^+^ and CD163^+^ cells in HD11 cells stimulated with LPS were significantly increased (*p* < 0.001). Pretreatment with PB1 significantly downregulated the proportion of MHC II^+^ cells and upregulated that of CD163^+^ cells in the LPS-stimulated HD11 cells (*p* < 0.05). Compared to the control without RA, RA treatment significantly elevated the proportion of MHC II^+^ cells and reduced that of CD163^+^ cells in both the LPS-alone and LPS + PB1 groups (*p* < 0.05) (Figure 7D). Conversely, in both the LPS + HIF1Ai and LPS + PB1 + HIF1Ai groups, the proportion of MHC II^+^ cells decreased significantly, while that of CD163^+^ cells increased significantly compared to their respective controls (the LPS and LPS + PB1 groups; *p* < 0.05). These results suggest that both STAT2 and HIF-1α serve as key functional targets through which PB1 modulates the polarization of LPS-stimulated HD11 cells.

## 4. Discussion

Rich in anthocyanins and other polyphenolic compounds, many plant tissue extracts and grape winemaking by-products are considered valuable for significantly improving animal immunity and antioxidant status [30,31]. *Cyperus* is an edible perennial plant, with studies showing that extracts from its tubers, stems, and leaves possess allelopathic, antibacterial, antioxidant, and insecticidal activities [32]. A recent study reported that procyanidin B1 (PB1), the predominant compound in ECLE, exhibited strong in vitro antioxidant activity and protected zebrafish from tebuconazole-induced developmental toxicity and hepatotoxicity [18]. Our chicken model study confirms CELE’s antioxidant capacity and further demonstrates that it significantly attenuates LPS-induced inflammation, indicating potent anti-inflammatory activity without imposing any measurable metabolic burden or toxic stress. These results are consistent with the findings of other studies on procyanidins in broilers [33]. We therefore investigated the transcriptional mechanisms of PB1, CELE’s primary component. While previous studies showed that grape seed procyanidin alleviates inflammation via STAT3, NF-κB, and TREM2/PI3K/Akt pathways [34,35], we newly identified STAT2 and HIF1A as key regulators mediating PB1’s anti-inflammatory effects.

STAT2, a key transducer in the interferon pathway, typically forms the ISGF3 complex with STAT1 and IRF9 to enter the nucleus and drive the expression of antiviral and inflammatory genes [36]. Upstream, Janus kinase (JAK)—a non-receptor tyrosine kinase activated by inflammatory factors like IL-6 and IFN-γ—regulates STAT1/STAT2 [37]. Studies indicate STAT2 loss reduces NF-κB target gene expression by impairing its nuclear translocation [38]. While unphosphorylated STAT2 can bind the IL-6 promoter to enhance its expression [39]. Thus, STAT1/STAT2 is hypothesized to play a predominant role in proinflammatory responses triggered by bacterial/LPS stimulation. STAT2 target genes EPSTI1 and IFIH1 regulate macrophage polarization and inflammation: EPSTI1 knockdown reduces LPS-induced injury and promotes M2 polarization by inhibiting STAT1/p65 nuclear translocation [40], while IFIH1 promotes M1 polarization [41]. In this study, PB1 treatment downregulated STAT2, EPSTI1, and IFIH1 transcription, reduced M1 polarization, and decreased ROS and proinflammatory factors in LPS-induced HD11 macrophages, indicating that PB1 exerts anti-inflammatory effects by inhibiting the STAT2 pathway and its target genes.

Previous studies have indicated that some Chinese herbal extracts can modulate inflammation via the STAT1/STAT2 pathway. For example, Astragaloside IV (AS-IV) binds to STAT1 and promotes its dephosphorylation at Tyr701, facilitating M2 macrophage polarization in inflammatory bowel disease [42]. Resveratrol inhibits JAK2 and STAT1 phosphorylation and suppresses STAT3 transcriptional activity by promoting its deacetylation via SIRT1 activation [43,44]. Baicalein significantly inhibits IFN-γ–induced STAT1 (Tyr701) and IL-6–induced STAT3 (Tyr705) phosphorylation, attenuates NF-κB activation, and exerts anti-inflammatory effects [45,46]. Procyanidins were shown to reduce M1 polarization and cytokine production by inhibiting STAT3 and NF-κB phosphorylation [47]. In this study, we found that PB1 suppresses M1 polarization and promotes M2 polarization in HD11 cells through a distinct mechanism. We identified STAT2 as a potential target of PB1, which transcriptionally suppresses STAT2 and its downstream genes to modulate LPS-induced M1 polarization and cytokine release.

Our study also identified HIF1α as a key target mediating the anti-inflammatory effect of PB1. HIF-1α promotes M1 polarization and proinflammatory responses by suppressing the mitochondrial Tricarboxylic acid (TCA) cycle, enhancing glycolysis, and inducing mitochondrial ROS production [48,49]. HIF-1α-deficient macrophages show reduced iNOS and TNF-α, increased IL-10, and impaired migration [50]. Downstream targets of HIF-1α, such as HMGA2 and STEAP4, also contribute to inflammation regulation. HMGA2 silencing inhibits NF-κB signaling and reduces IL-6 and IL-8 expression, alleviating LPS-induced cytotoxicity [51]. Moreover, this silencing reduces LPS-induced damage to cell viability, thereby decreasing inflammation and apoptosis [52]. STEAP4, regulated in a hypoxia-dependent manner, disrupts mitochondrial iron homeostasis and promotes ROS generation; its knockdown mitigates LPS-induced inflammation [53]. Multiple active compounds, including Baicalin and Astragaloside IV, exert anti-inflammatory and antioxidant effects via HIF-1α and its targets [54,55].In our study, PB1 downregulated HIF-1α transcription, its target genes, proinflammatory factors, ROS levels, and M1 markers in LPS-treated HD11 cells, indicating that HIF-1α is an important target through which PB1 exerts anti-inflammatory activity.

Macrophage polarization and reactive oxygen species (ROS) production are regulated by multiple signaling pathways. TLR4/NF-κB represents the canonical pathway for macrophage polarization to the M1 phenotype. M1-polarized macrophages generate ROS through NADPH oxidase (NOX) and mitochondrial electron leakage. As mentioned earlier, NOX-mediated ROS is regulated by HIF1α. Furthermore, the amplification of NF-κB signaling and the M1 inflammatory response relies on IFN-γ-mediated activation of the JAK1/2–STAT1/2 pathway [39,56]. Our findings thus suggest that Stat2 and HIF1α are potential targets underlying PB1-mediated regulation of macrophage polarization and ROS production.

However, a limitation of this study is that we employed a crude extract of *Cyperus esculentus* rather than purified procyanidin B1. The *Cyperus esculentus* extract contains a variety of bioactive components, including, but not limited to, other flavan-3-ols (e.g., gallocatechin), caffeic acid derivatives (e.g., chlorogenic acid), and flavones (e.g., orientin) [22]. Consequently, the anti-inflammatory and antioxidant effects we observed are likely the result of the combined action of these constituents, implying the existence of additive or synergistic effects. Although procyanidin B1 was identified as one of the major active components in our extract, the extent of its individual contribution remains to be elucidated. Future studies will utilize purified procyanidin B1 and other isolated compounds in comparative experiments to precisely delineate the contribution of each component and its mechanisms of interaction.

## 5. Conclusions

In conclusion, our study demonstrates that the dietary supplementation of CELE, enriched with procyanidin B1 (PB1), effectively alleviates oxidative stress and modulates inflammatory responses in chicken layers. The underlying mechanism is potentially associated with the polarization of macrophages towards the M1 and M2 phenotypes via the STAT2 and HIF1A signaling pathways. Beyond these findings, our research provides practical implications for the use of polyphenols in poultry farming. Specifically, we recommend considering this PB1-enriched extract as a potential functional feed additive in the following scenarios: (i) For alleviating oxidative stress and inflammation associated with common production diseases such as necrotic enteritis and dysbiosis; (ii) as a supportive nutritional strategy during periods of high immunological challenge, like vaccinations or environmental stressors, to maintain immune homeostasis; (iii) potentially, as part of a strategy to reduce the reliance on in-feed antibiotics, given its anti-inflammatory and antioxidant properties.

## Data Availability

The data generated in this study are available in the NCBI GEO database under accession number GSE309607 (access token: olmpcgqgthurnib).

## Supplementary Materials

The following supporting information can be downloaded at: https://www.mdpi.com/article/10.3390/ani15233469/s1, Table S1: Primers for Realtime-PCR; Table S2: Differential expression lists; Figure S1: Typical HPLC-UV chromatograms of Cyperus Esculentus stem and leaf extract (A), and the Procyanidin B1 standard (B), acquired at 210 nm.

## Abbreviations

The following abbreviations are used in this manuscript:

PB1	Procyanidin B1
GenAI	Generative artificial intelligence
STAT 1	Signal transducer and activator of transcription 1
STAT 2	Signal transducer and activator of transcription 1
STAT 3	Signal transducer and activator of transcription 3
HIF-1	Hypoxia-inducible factor-1
FOSL 1	Fos-Related Antigen 1
SOD	Superoxide Dismutase
