# Supplementary material for: Procyanidin-B1-Enriched Cyperus esculentus Extract Regulates Anti-Inflammatory Pathways in Chicken Macrophages Cells Through Transcription Factor STAT2 and HIF1A
Source: Animals (Basel). 2025 Dec 2;15(23):3469. doi: 10.3390/ani15233469 (PMC12691537; doi:10.3390/ani15233469)
Supplement: Supplementary file 1 [file animals-15-03469-s001.zip › Supplemental Document.pdf]

Supplemental Document

Extraction of the stems and leaves of Cyperus Esculentus

To each 100 g of dried *Cyperus esculentus* stem-leaf powder, add 2 L of 70% methanol solution at a solid-to-liquid ratio of 1:20. After soaking overnight, sonicate the mixture (600 W, 40 kHz) for 30 minutes, followed by centrifugation at 5000 rpm for 10 min. Filter the supernatant through a 0.22 μm microporous membrane. The filtrate was applied to a Sephadex LH 20 column eluted with ethanol. Collected the fractions that displayed a single peak of procyanidin B1 in HPLC and freeze-dried to dryness to obtain the crude extract, which is subsequently redissolved in water for HPLC-UV analysis.

HPLC apparatus and analytical conditions

Twenty microliters of *Cyperus esculentus* stem and leaf extract were injected into an HPLC system (Waters 2695) equipped with an Agilent ZORBAX Eclipse XDB-C18 column (250 × 4.6 mm, 5 μm). The mobile phase consisted of acetonitrile–water (12:88, v/v) containing 0.1% formic acid, delivered at a flow rate of 1 mL/min. Chromatograms were monitored at 210 nm. Compounds were identified by comparison of their retention times with that of a procyanidin B1 reference standard. Quantification was achieved by interpolating peak areas measured at 210 nm into the corresponding calibration curves.

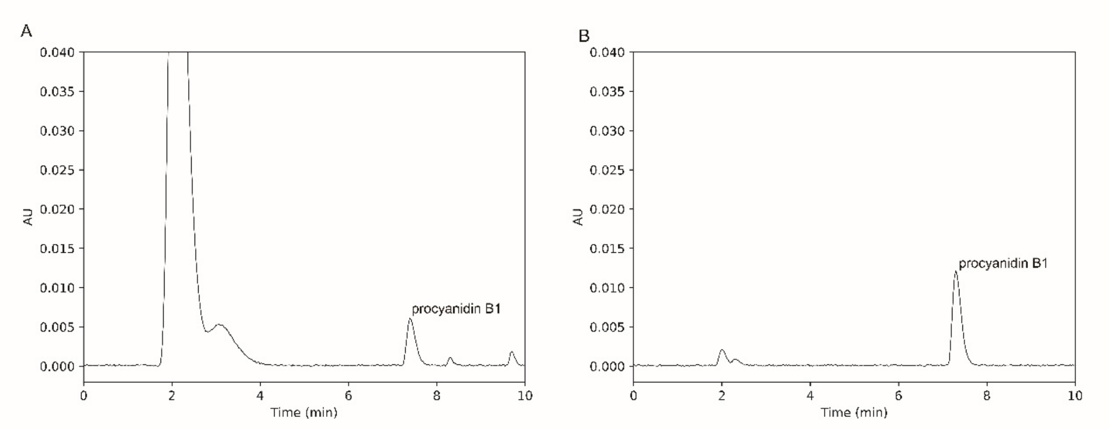

Figure S1. Typical HPLC-UV chromatograms of Cyperus Esculentus stem and leaf extract (A), and the Procyanidin B1 standard (B), acquired at 210 nm.
